# Oral Cavity Metastasis of Hepatocellular Carcinoma following Liver Transplantation

**DOI:** 10.1155/2012/181242

**Published:** 2012-10-04

**Authors:** Nicolás Goldaracena, Mariela Barreto, Gabriel Casas, Margarita Anders, Ricardo Mastai, Lucas McCormack

**Affiliations:** ^1^Liver Surgery and Transplantation Unit, Hospital Alemán of Buenos Aires, Avenne Pueyrredón 1640, Buenos Aires 1118AAT, Argentina; ^2^Pathology Service, Hospital Alemán of Buenos Aires, Avenne Pueyrredón 1640, Buenos Aires 1118AAT, Argentina; ^3^Hepatology Service and Liver Transplantation Unit, Hospital Alemán of Buenos Aires, Avenne Pueyrredón 1640, Buenos Aires 1118AAT, Argentina

## Abstract

Liver transplantation (LT) remains the only cure for hepatocellular carcinoma (HCC) in many cases. Over many years, most centers have applied the Milan criteria for selecting cirrhotic patients with HCC for LT. In a new era where several transplant groups are pushing the limits of transplanting HCC outside Milan criteria as an aggressive approach with promising results, we present interesting images of a patient that presented a unique and rare site of HCC metastasis after 36 months of liver transplantation.

## 1. Introduction

Over many years, most centers have applied the Milan selection criteria for cirrhotic patients with hepatocellular carcinoma (HCC). However, several groups from different countries have challenged these restrictive criteria with promising results [1]. This more aggressive approach could result in higher rates of tumor recurrence that should carefully be assessed in these patients with advanced HCC within the liver. We report here a case with an unusual site of tumor recurrence after liver transplantation (LT) that should be known as a potential site of metastasis.

## 2. Case Report

A 58-year-old male patient with liver cirrhosis due to hepatitis C virus (HCV) infection and alcohol consumption with HCC beyond Milan criteria underwent LT at our unit. The pathological study of the liver specimen revealed multiple well-differentiated HCC with a major nodule of 6,6 cm in diameter without vascular invasion (T3aN0M0). After LT the patient received tacrolimus and mycophenolate as immunosuppression regimen. During follow-up, he never experienced HCV recurrence. In addition, any episodes of rejection or opportunistic infections needing specific treatment were detected.

**Figure 1:**
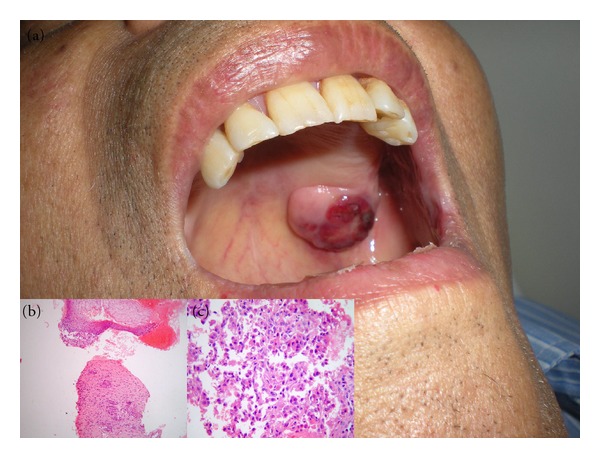
(a) Patient oral cavity showing 2 cm exophytic mass in the left retromolar trigonum; biopsy from the oral mass (hematoxylin and eosin stain) demonstrates infiltration by carcinoma of nodular and glandular pattern, consisting of atypical cuboidal cells with slightly acidophilic granular cytoplasm. (b) Magnification × 40. (c) Magnification × 100.

**Figure 2:**
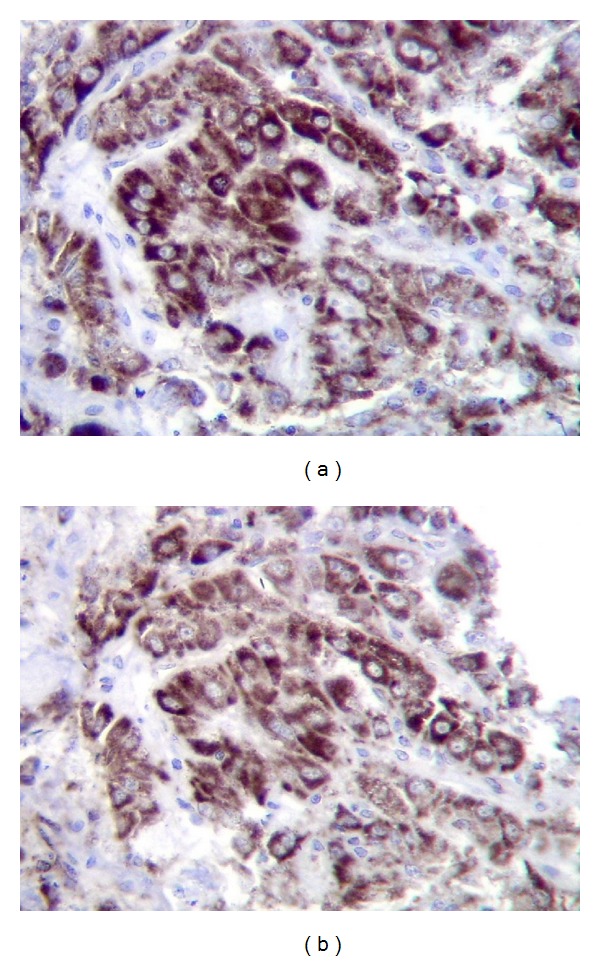
Immunohistochemistry showing expression of Heppar 1 and CD34 on the tumor biopsy: (a) expression of Heppar 1 (magnification × 400) and (b) expression of CD34 (magnification × 400).

Follow-up images and tumoral markers showed no tumoral recurrence until 36 months after LT when he was admitted to our hospital presenting with a 2 cm in diameter bleeding oral mass localized in the retromolar trigonum (Figure 1(a)). The bleeding stopped by local compression, and a biopsy of the lesion was taken. The pathological study of the oral lesion demonstrated mucosal infiltration by a poor-differentiated carcinoma (Figures 1(b) and 1(c)). Immunohistochemistry was performed and the lesion was positive for Heppar 1 and CD34 (Figure 2). With the morphological features and the immunohistochemical profile the oral lesion was diagnosed as a metastatic HCC.

After diagnosing the oral lesion as a metastatic HCC a PET-CT was performed showing no other metastatic disease elsewhere. Alpha-fetoprotein levels were still normal. His immunosuppression was switched to sirolimus, and he started treatment with sorafenib 400 mg/12 hs.

## 3. Discussion

To the best of our knowledge, this is the first case of a metastatic HCC to the oral cavity after LT reported in English literature. Surprisingly, this was the only site of metastasis after 36 months of LT due to HCC beyond Milan criteria.

In patients transplanted due to HCC on a cirrhotic liver the most frequent site of metastasis is the transplanted liver. Other sites in which HCC metastases most frequently occur are the lungs, adrenal glands, and bones. Interestingly, the PET-CT did not show any metastasis elsewhere, assuming that its long-term recurrence first appeared at a distant location and not in the new liver, as expected. Metastases from HCC to the oral cavity have only been reported occasionally but only in patients with disseminated disease that did not receive a LT [2].

Most cases of metastatic HCC in the oral cavity involve the mandible and gingival presenting either as exophytic tumors or as intraosseous lesions [3]. Usually, the symptoms caused by this type of metastatic lesions are pain, dysphagia, and bleeding among others. In our case, the oral metastasis presented as a gingival exophytic bleeding mass without bone involvement. 

When oral metastasis appears, the prognosis is very poor. therefore these lesions are treated with surgery, radiotherapy, or sorafenib to relieve symptoms and improve the quality of life [4]. Unfortunately, in the setting of a patient with immunosuppression with sirolimus after LT, data is lacking in the literature. 

## Abbreviations

HCC: Hepatocellular carcinoma
LT: Liver transplantation
HCV: Hepatitis C virus.
